# Baths and Bathing

**Published:** 1861-02

**Authors:** 


**BATHS AND BATHING.**—A cold bath is 75° and under; temperate, 75° to 85°; tepid, 85° to 95°; warm, 95° to 100°; hot, 100° and over.

The temperature of the body in health is ninety-eight degrees Fahrenheit. For purposes of cleansing the skin, a hot bath is the most efficient, but it should be indulged in only occasionally, and for a very few minutes at a time, as it rapidly exhausts the physical powers. It opens the pores of the skin and increases the activity of the circulation for the moment, but if followed by an instantaneous cold shower-bath, an invigorating effect is produced. A hot bath excites, a warm bath soothes and tranquillizes; it makes the pulse slower, and causes more equable breathing.

A vapor-bath is of steam instead of water, and is applied inside as well as out; its first effect is a feeling of oppression, but soon perspiration is induced, and delightful sensations ensue. To prevent taking cold, the person should pass from the steam-chamber into a tepid bath for a single moment, then wipe dry briskly, dress and walk.

No kind of bath ought to be taken within an hour before a regular meal, nor sooner than four hours after; sudden death has often resulted from inattention to the latter. The best time for bathing is immediately after rising in the morning, as then there is greater power of reäction, without which there is no invigoration, no benefit.

The sponge-bath is the application of water to the surface of the body by means of a sponge. When persons are feeble, one portion of the body should undergo the process at a time, then quickly wiped and dried, and covered, before another is exposed. There are few persons indeed who would not be greatly benefited by the following procedure every morning, winter and summer: Wash the hands first in a small amount of water with soap, for if but little is used, a teacupful, it is warmed by the hands, and thus becomes more cleansing, without the trouble of preparing warm water; then rinse them well; afterwards wash the face in a large basin of cold water just drawn or brought into the room, for all cold water becomes filthy in an hour or two if kept standing in a sitting or sleeping apartment. After the face has been washed plentifully, throw the water up to the elbows, then a little higher at every dash with the hand, until the arms, neck, throat, behind the ears, arm-pits, and upper portion of the chest have been deluged with water; next (except women with long hair) wash the whole scalp abundantly, rubbing the water into and about the roots of the hair with the ends of the fingers; then wipe with a towel, absorbing as much of the dampness from the hair as possible with an extra dry cloth, and dress, leaving the arrangement of the hair to the last, so as to give it an opportunity of drying somewhat; for if it is wringing wet, it will not dress well, and besides will keep the head cold by its evaporation. In dressing the hair after such a washing of the head, the comb should be passed through it in the gentlest manner, so as to make no strain upon the roots, nor break any hair in disengaging the tangles. The hair thus dressed in the morning will remain so the whole day, or, if not, can easily be re-dressed, with the advantage of perfect cleanliness, which can not be said of the filthy practice of using hair-oils.

